# Feasibility of quality of life assessment in patients with upper gastrointestinal tract cancer

**DOI:** 10.1038/sj.bjc.6601146

**Published:** 2003-07-29

**Authors:** J M Blazeby, J Nicklin, S T Brookes, K Winstone, D Alderson

**Affiliations:** 1 University Division of Surgery, Bristol Royal Infirmary, Bristol BS2 8HW, UK; 2 Department of Social Medicine, University of Bristol, Bristol BS8 2PR, UK

**Keywords:** quality of life, EORTC QLQ-C30, compliance, upper gastrointestinal cancer

## Abstract

Quality of life (QOL) is an important outcome after treatment for upper gastrointestinal tract cancer but few studies report good accrual and subsequent attrition is usually high. This study investigated the feasibility of a nurse-led service to obtain longitudinal QOL assessments and explored how clinical and sociodemographic factors influence patients' need for help to complete questionnaires. Fully informed patients were invited into the study. Baseline hospital assessments were scheduled by telephone and thereafter by post unless patients' health indicated the need for a home visit. In all, 128 out of 140 (91%) baseline QOL assessments were performed. Follow-up questionnaire completion was good, with 114 patients (89%) completing all but one of the expected assessments. At baseline, 41 (32%) patients required a lot of help to complete questionnaires. Patients requiring help were more likely to be undergoing palliative treatment than treatment aimed at cure (68 *vs* 33%; odds ratio 3.48, *P*<0.01). Patients' with advanced stage cancer of the upper gastrointestinal tract receiving palliative treatment require dedicated staff to ensure good compliance with longitudinal QOL data collection. It is essential to budget for this in clinical trails.

The American Society of Clinical Oncology recommends that assessment of patient outcomes (e.g., survival and quality of life) is more important than other outcomes (e.g., tumour response rates and biomarkers) in the evaluation of new technologies and in development of cancer treatment guidelines (The American Society of Clinical Oncology, 1996). Survival data are routinely collected in most clinical trials and many are now including quality of life (QOL) as a secondary end point. Interest in assessing QOL is also increasing in routine clinical practice (Detmar and Aaronson, 1998). Quality of life data are valuable end points because they monitor change in patient-reported symptom experience and functional health over time and there is evidence to show that they facilitate communication between doctors and patients (Detmar *et al*, 2000). For patients with cancer of the upper gastrointestinal tract (oesophagus and stomach), these considerations are especially important, because the disease and treatment (surgery, endoscopic palliation or chemoradiation therapy) may have significant morbidity and impact on QOL. Studies attempting to measure changes in QOL over time in patients with upper gastrointestinal cancer, have however, frequently reported poor accrual and high attrition rates with QOL assessments (Barr and Krasner, 1991; Webb *et al*, 1997; Ilson *et al*, 1999; Ross *et al*, 2002). These reports often lack details of QOL methods used to collect data (interview administered or self-completion, reasons for nonparticipation and attrition). Poor compliance and reporting of QOL data in clinical trials may bias the results and invalidate conclusions (Nordin *et al*, 2001).

Efforts to improve compliance with QOL assessment have mostly focused on developing psychometrically robust tools and less about the practical details of administering questionnaires. Computer-based assessments in outpatient clinics may be used (Velikova *et al*, 2002), but this still requires patients to attend the hospital. In view of the importance of accurate QOL data to help define treatment for upper gastrointestinal tract cancer, this study was undertaken with the following aims: to describe a method of obtaining good compliance with longitudinal QOL measurements from patients with oesophageal and gastric cancer and to examine factors that determine whether patients need help to complete questionnaires.

## METHODS

Between November 2000 and November 2001 consecutive new patients presenting to one upper gastrointestinal unit, with oesophageal or gastric cancer were considered eligible to participate in a longitudinal study evaluating the impact of treatment for oesophageal and gastric cancer on QOL. Patients were excluded if they were unable to understand the language of the questionnaire, if they had brain metastases, delirium or confusion or if they had other previous or concurrent malignancies. There were no limits on age or performance status. Patients were identified at the upper gastrointestinal cancer multidisciplinary meeting where all new patients are discussed. All new patients were informed of the diagnosis and an assessment of general health was performed before undergoing staging investigations. Staging investigations included chest and abdominal computerised tomography, selective endoluminal ultrasonography and where indicated laparoscopy. Patients were then rediscussed at the multidisciplinary meeting and a treatment plan was recommended. The plan was discussed with the patient in clinic. Informed patients, therefore, aware of the diagnosis and proposed treatment were invited to take part in this QOL study. Patients were informed that the purpose of the study was to assess how the disease and treatment impacts on specific aspects of their health. They were aware that they would be asked to fill in repeated questionnaire assessments. Clinical and sociodemographic data were recorded. Ethics committee permission and written informed consent were obtained.

### Quality of life assessments

Two part time research nurses coordinated data collection. Baseline interviews were arranged by telephone. Patients attending the hospital within the time frame for the first assessment underwent baseline assessment in hospital. Occasionally, patients who were able were asked to complete postal baseline assessments. Patients who could not be interviewed in hospital before the start of treatment and those who did not understand the nature of the research on the telephone were visited at home for their first QOL assessment. During the first QOL assessment, the nurse obtained informed consent. Patients were then asked to complete questionnaires themselves. Patients who asked for help at this point either were encouraged to continue with questionnaire self-completion or questionnaires were interview administered. Time taken to complete questionnaires and the degree of help required was recorded. The degree of help was categorised into no help, a little help (brief explanation of a few items), quite a bit (help with almost all items) or a lot of help (the questionnaire was read to the patient).

### Follow-up assessments

Patients who had required a lot of help at the baseline assessment to complete the questionnaires continued to receive this degree of support during follow-up. Other follow-up assessments were arranged by telephone and performed by post. Patients with declining health, not due to attending the hospital or those with a poor prognosis, were offered a home visit. Patients failing to return postal questionnaires received one telephone call reminder. Reasons for withdrawing from QOL follow-up were recorded. Follow-up QOL assessments continued to be offered even after diagnosis of recurrent or progressive disease unless the nurses felt that it was inappropriate because of the patient's deterioration.

### Timing of QOL assessment

**Figure 1:**
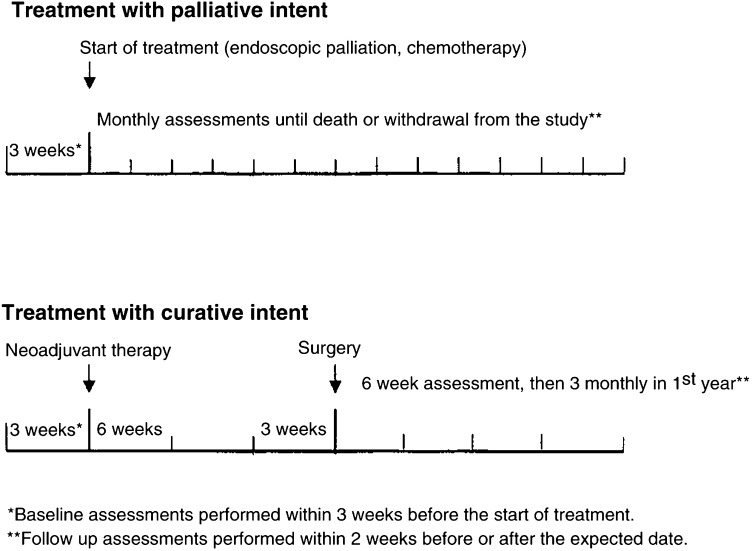
Timing of QOL assessment per treatment protocol. ^*^Baseline assessments performed within 3 weeks before the start of treatment.

Baseline data were collected within 3 weeks before treatment and follow-up data were collected within 2 weeks either side of scheduled assessments. The schedule for follow-up QOL assessments varied according to treatment protocol. These are shown in Figure 1

.

### Quality of life questionnaires

Quality of life was assessed with the European Organisation for Research and Treatment of Cancer (EORTC) core QOL questionnaire, the EORTC QLQ-C30 and a site-specific module for oesophageal or gastric cancer (Aaronson *et al*, 1993). The QLQ-C30 has been reported in detail elsewhere and has been validated in patients with many diagnoses including cancer of the upper gastrointestinal tract. The oesophageal (QLQ-OES18) and gastric (QLQ-STO22) modules have undergone strict questionnaire development and are widely used (Blazeby *et al*, 1996; Vickery *et al*, 2001). The QLQ-OES18 has recently completed international validation testing (Blazeby *et al*, 2003) and the QLQ-STO22 is currently being validated in EORTC protocol 15012.

### Data analyses

Patients were followed until death or the end of the study period (September 2002) when all surviving patients were censored. The actual and expected numbers of QOL assessments obtained were calculated for all patients according to the data collection schedule (Figure 1) and survival time. Follow-up assessments declined by patients within 6 weeks of death were not included as an expected assessment. The impact of clinical and sociodemographic factors on the need for help to complete questionnaires was explored using logistic regression. Marital status was not included because it closely linked to living situation. Gender, living situation and treatment group were dichotomised (male, yes/no; living alone, yes/no, curative treatment, yes/no). Education was collapsed to three variables and age was included as a continuous measure. Data were analysed in STATA version 7.0 and SPSS version 10.00.

## RESULTS

In total, 141 patients were eligible to take part in the QOL study. A total of 12 (9%) did not participate for the following reasons: four patients' relatives felt that it was inappropriate, two patients were too upset, three said that they were too ill to complete questionnaires and two refused without explanation. The nurse did not approach one patient because his wife died the preceding week. Three patients not participating in the study were embarking on neoadjuvant chemotherapy prior to surgery, eight were receiving treatment aimed at palliation (endoscopic stent or chemotherapy) and one was receiving best supportive care. Complete baseline QOL assessments were therefore obtained from 128 patients (91%), 90 and 38 with oesophageal and gastric cancer, respectively.

### Baseline QOL assessment

At the baseline assessment, 77 (60%) patients were able to complete the questionnaires without any help from the research nurse. A total of 10 patients required minor explanation of one or two items. These 87 patients were grouped together as not requiring help (Group 1). The remaining 41 patients formed Group 2. This included patients needing a lot of help from the nurse to complete it themselves (*n*=7) and patients who required the questionnaire read aloud (*n*=34). The time taken to complete the questionnaires was similar in both groups of patients, and sociodemographic and clinical details were similar except for treatment group (Tables 1

**Table 1:** Clinical and sociodemographic details of patients needing help to complete baseline questionnaires

	**No help (Group 1) *n*=87**	**Help (Group 2) *n*=41**
Mean age (standard deviation)	67 (10.5)	71 (11.6)
Gender		
Men (%)	66 (76)	29 (71)
Women	21 (24)	12 (29)

Karnofsky performance status		
Mean (standard deviation)	81 (12)	73 (13)

Living situation		
Living with others (%)	18 (21)	12 (29)
Living alone	69 (79)	29 (71)

Highest level of education (%)		
Less than school	8 (9)	9 (22)
Compulsory school	58 (67)	24 (59)
Post compulsory school	21 (24)	8 (19)

Diagnosis		
Oesophageal cancer	59 (68)	31 (76)
Gastric cancer	28 (32)	10 (24)

Time taken to complete questionnaires (%)		
<15 min	68 (78)	31 (76)
16–30 min	15 (17)	9 (22)
>30 min	4 (5)	1 (2)

Undergoing radical treatment (total)	(58) (67%)	(13) (32%)
Oesophagectomy or radical gastrectomy	24	4
Neoadjuvant treatment and surgery	30	9
Primary chemoradiotherapy	4	—

Undergoing palliative treatment (total)	(29) (33%)	(28) (68%)
Palliative chemotherapy	12	7
Endoscopic palliation	8	12
Palliative bypass surgery	5	2
Best supportive care	4	6

Compliance with follow-up (%)		
0 missing assessments	77 (89)	37 (90)
⩾1 missing assessments	10 (11)	4 (10)

and 2

**Table 2:** Logistic regression model examining factors relating to needing help to complete QOL questionnaires

**Variables**	**Odds ratio**	**Confidence intervals**	***P*-value**
Age (per year increase)	1.01	0.97–1.05	0.72
Gender (women *vs* men)	1.42	0.57–3.55	0.46
Treatment intent (palliative *vs* curative)	3.48	1.41–8.55	<0.007
Performance status (per unit increase)	0.97	0.94–1.00	0.088

).

Multivariable logistic regression adjusting for age and gender demonstrated that there was moderate evidence that the lower the performance score the more likely that help would be required (for a drop in performance score of 1 point, odds ratio=1.03; for a drop of 10 points, odds ratio=1.34, that is a 34% increase in odds for needing help). Moreover, patients undergoing treatment with palliative intent were nearly three and a half times more likely to need help than those undergoing potentially curative treatment (odds ratio=3.48, 95% confidence intervals 1.41–8.55) (Table 2).

### Follow-up

In all, 80 died during follow-up. The median survival for whole group was 45 weeks (range, 1 day to 97 weeks). During follow-up, 810 QOL assessments were performed from an expected total of 834 (97%). Only 14 (11%) patients had one or more missing QOL assessment. These were mostly because of progressive disease (*n*=11). One patient with a large alcohol intake refused follow-up, one patients' wife requested to drop out of the study and one patient refused follow-up without explanation. Compliance with follow-up was better in patients receiving help from the nurses to complete questionnaires (Table 1).

Notably of 62 patients who were phoned for a follow-up QOL assessment within 6 weeks of subsequent death, 48 (77%) agreed to return a questionnaire or be visited at home.

Questionnaires in the curative treatment group were initially administered by post in all except two patients (one was blind). Patients who developed recurrence during the study were offered home visits for QOL assessments. Of patients whose treatment was palliative in intent (who mostly needed a lot of help at baseline) all had nurse-led follow-up assessments except three patients who completed postal assessments.

## DISCUSSION

This study demonstrates that a nurse-led service to collect longitudinal QOL data may achieve high baseline QOL compliance and good follow-up in patients with upper gastrointestinal tract cancer. Patients undergoing potentially curative treatment may have QOL questionnaires administered by post with additional telephone reminders. Patients with advanced disease, however, or those not fit for radical treatment are more likely to need help to complete QOL questionnaires and require an interview administered assessment, either in the hospital or at the patients' home. Patients with poor performance scores may need help to complete questionnaires, although in this study sample this did not reach statistical significance.

The EORTC QLQ-C30 and a site-specific module assessed QOL in this study. These instruments provide multidimensional QOL data, but patients are required to complete more than 50 items. Others advocate using global single-item QOL indicators to implement QOL end points in cancer clinical trials (Sloan, 2002). This approach has clear cut advantages, but may yield less reliable data and reduce discriminant validity (Bernhard *et al*, 2001). Other disadvantages of single-item QOL indicators are that they are less precise for specific treatment effects than multiitem scales. Detailed information about patients undergoing treatment with curative intent is useful to monitor symptoms and aid patient care. Detailed information about specific problems during palliative treatment is also important to ensure that care is targeted appropriately. Other QOL questionnaires designed specifically for palliative care may include existential well being and spiritual domains, but there are no single measures that cover physical, psychological and spiritual domains in a format that will provide sufficient and reliable information (Hearn and Higginson, 1997). In addition, few measures include an assessment of dysphagia, which is an important symptom to assess in patients with cancer of the upper gastrointestinal tract. The EORTC oesophageal and gastric modules include this and other relevant symptoms that most clinicians consider important in evaluation of treatment (Blazeby *et al*, 1996; Vickery *et al*, 2001; Blazeby *et al*, 2003).

In this single-centre study, repeated QOL assessments were performed successfully in nearly 90% of patients. This is unusual, but because of the design of the study it is not possible to know how a nurse-led QOL service compares with other methods of QOL data collection. A few authors have reported high accrual with longitudinal QOL assessment in upper gastrointestinal cancer. Buhl *et al* reported good compliance by using a semistructured interview in all assessments. A more recent small study comparing laser augmented by brachy therapy *vs* laser alone in palliation of adenocarcinoma of the oesophagus also had good QOL accrual (Spencer *et al*, 2002). The authors attributed this to the brevity of the QOL questionnaire. There are no details available, however, about how questionnaires were administered and who collected the data. Such information is required when reporting QOL data particularly from a population with a poor prognosis as well as other important methodological criteria for assessing QOL (Lee and Chi, 2000). In this current study, patients' willingness to continue with questionnaire completion may be attributed to the expertise of the research nurses who were able to provide both specialist advice (often nutritional) and psychological support for the patient and family during the course of the illness.

In addition to achieving a high level of follow-up, QOL assessments were performed in 77% of patients within 6 weeks of their subsequent death. This testifies to the good relationships that the research nurses had developed with the patients who were happy to complete questionnaires despite being frail and unwell. There is also some evidence to suggest that completion of QOL questionnaires is beneficial to the patients *per se* because it empowers patients to talk about aspects of their general health that are not usually discussed during follow-up (Velikova *et al*, 2002). This study achieved good follow-up questionnaire compliance, but because of the design of the study, it is not possible to conclude that nurses are essential to ensure high compliance with QOL data.

The standard of reporting clinical trials is fundamental to the interpretation of individual studies and the conduct of systematic reviews. Guidelines have been published for assessing QOL in clinical trials from several organisations (De Haes *et al*, 2000; Kiebert *et al*, 2001). This provides investigators with practical information. Despite these guidelines, improvements are still needed in QOL data collection and reporting (Sanders *et al*, 1998; Lee and Chi, 2000). This study provides evidence to support the need for dedicated research staff to obtain QOL data in clinical trials in upper gastrointestinal tract cancer. If assessment of QOL is the primary end point in a clinical trial such investment is essential, and for trials where QOL is a secondary end point careful consideration as to how QOL should be incorporated into the trial is necessary.
